# Structural Insights into the Binding Propensity of Human SHIP2 SH2 to Oncogenic CagA Isoforms from *Helicobacter pylori*

**DOI:** 10.3390/ijms231911299

**Published:** 2022-09-25

**Authors:** Zi Wang, Yubao Shan, Ru Wang, Heng Zhou, Rui Hu, Ying Li, Jiang Zhu, Yunhuang Yang, Maili Liu

**Affiliations:** 1 State Key Laboratory of Magnetic Resonance and Atomic Molecular Physics, Key Laboratory of Magnetic Resonance in Biological Systems, National Center for Magnetic Resonance in Wuhan, Wuhan Institute of Physics and Mathematics, Innovation Academy for Precision Measurement Science and Technology, Chinese Academy of Sciences—Wuhan National Laboratory for Optoelectronics, Wuhan 430071, China; 2 University of Chinese Academy of Sciences, Beijing 100049, China

**Keywords:** NMR, INPPL1, SH2 domain, tyrosine phosphorylation, protein–protein interaction

## Abstract

SHIP2 is a multi-domain inositol 5-phosphatase binding to a variety of phosphotyrosine (pY)-containing proteins through its SH2 domain, so as to regulate various cell signaling pathways by modulating the phosphatidylinositol level in the plasma membrane. Unfavorably, *Helicobacter pylori* can hijack SHIP2 through the CagA protein to induce gastric cell carcinogenesis. To date, the interaction between SHIP2 and CagA was not analyzed from a structural point of view. Here, the binding of SHIP2-SH2 with Tyr-phosphorylated peptides from four EPIYA motifs (A/B/C/D) in CagA was studied using NMR spectroscopy. The results showed that EPIYA-C and -D bind to a similar interface of SHIP2-SH2, including a pY-binding pocket and a hydrophobic pocket, to achieve high affinity, while EPIYA-A and -B bind to a smaller interface of SHIP2-SH2 with weak affinity. By summarizing the interface and affinity of SHIP2-SH2 for CagA EPIYA-A/B/C/D, c-MET and FcgR2B ITIM, it was proposed that, potentially, SHIP2-SH2 has a selective preference for L > I > V for the aliphatic residues at the pY+3 position in its ligand. This study reveals the rule of the ligand sequence bound by SHIP2-SH2 and the mechanism by which CagA protein hijacks SHIP2, which will help design a peptide inhibitor against SHIP2-SH2.

## 1. Introduction

Inositol phospholipids (PtdIns) anchoring in the inner leaflet of the plasma membrane are critical molecules mediating various cell signaling cascades [1]. Inositol kinases and inositol phosphatases modify PtdIns to regulate signaling cascades that result in cell proliferation, differentiation, or migration [2,3]. Therein, Src homology 2 (SH2) domain-containing inositol 5-phosphatase 2 (SHIP2, also named INPPL1—inositol polyphosphate phosphatase like 1) hydrolyzes the 5-phosphate of phosphatidylinositol-3,4,5-trisphosphate (PI(3,4,5)P3) to produce PI(3,4)P2, and thus modulates the levels of PI(3,4,5)P3 and PI(3,4)P2, which affect the functions of numerous proteins in diverse cell signaling pathways [4,5]. Dysfunction of SHIP2 is associated with cancers, autoimmune diseases and Alzheimer’s disease (AD) [6,7,8,9,10].

SHIP2 is a multi-domain protein with the 5-phosphatase domain in the middle and several regulatory domains including SH2 and PH-R located in the N-terminal part of the protein, and C2, proline-rich, and the sterile alpha motif (SAM) located in the C-terminal part [11,12,13,14,15]. The SHIP2 SH2 domain (hereafter represented by SHIP2-SH2) can mediate the interaction of SHIP2 with a variety of proteins, such as p130cas [16], hepatocyte growth factor receptor (HGFR/c-MET) [17] and immunoreceptors [18,19], through binding to the phosphotyrosine (pY)-containing motifs in these proteins, so as to recruit SHIP2 to the corresponding pY-dependent signaling pathways. SHIP2-SH2 adopts a canonical SH2 structure consisting of six β-strands (βA to βF) flanked by two α-helices (αA and αB). A positively charged pY-binding pocket formed by αA, βB, βC, βD and the BC-loop (between βB and βC) can be found in the structure [13]. Although the pY-motifs in the native interaction partners of SHIP2-SH2 identified to date show low sequence similarity, a common characteristic can be found in that they all have aliphatic residues at the pY+3 positions [17,18,19,20]. Correspondingly, an electroneutral region adjacent to the pY-pocket is formed by βD, βE, αB, the EF-loop (between βE and βF) and the BG-loop (between αB and the C-terminus), potentially for accommodating hydrophobic residue [21].

**Figure 1:**
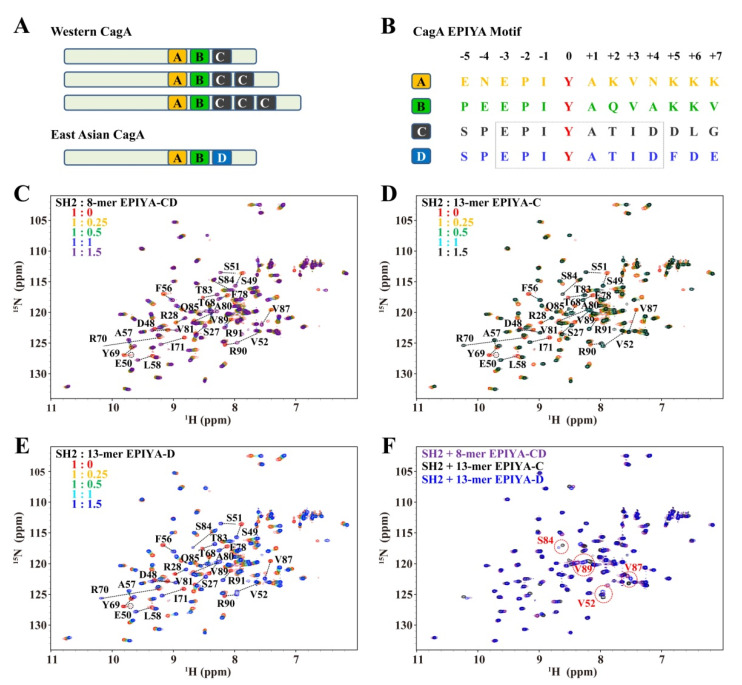
SHIP2-SH2 interacts with Tyr-phosphorylated CagA EPIYA-C and EPIYA-D motifs. (**A**) Domain diagrams of Western and East Asian CagA. (**B**) Amino acid sequences of four EPIYA motifs. The phosphorylated tyrosine is shown in red color. NMR titrations of SHIP2-SH2 by 8-mer Tyr-phosphorylated CagA EPIYA-CD peptide (**C**), 13-mer Tyr-phosphorylated CagA EPIYA-C peptide (**D**) and 13-mer Tyr-phosphorylated CagA EPIYA-D peptide (**E**). The residues with remarkable chemical shift perturbations are labeled in (**C**–**F**). (**F**) Superposed spectra of SHIP2-SH2 bound by Tyr-phosphorylated 8-mer EPIYA-CD peptide, 13-mer EPIYA-C peptide and 13-mer EPIYA-D peptide. The residues with chemical shift differences larger than 0.15 ppm are highlighted by red circles.

SHIP2-mediated PtdIns signaling can be hijacked by cytotoxin-associated antigen A (CagA)-positive *Helicobacter pylori*, a pathogen associated with the development of chronic gastritis, gastric ulcers, duodenal ulcers and gastric cancer [22,23]. CagA is a cytotoxicity-associated protein of *H. pylori*, and is injected into gastric epithelial cells by the bacterial type IV secretion system and then binds to the inner leaflet of the plasma membrane, where it is phosphorylated by host kinases at the Tyr residue in the Glu-Pro-Ile-Tyr-Ala (EPIYA) motif [24,25]. The phosphorylation enables CagA to interact with host SH2 domain-containing proteins and utilize their functions [22,26,27,28]. Four types of EPIYA motifs (EPIYA-A, EPIYA-B, EPIYA-C and EPIYA-D), differing in the sequence following the Ala residue, are present in various numbers and combinations in CagA from different *H. pylori* strains [29]. CagA in *H. pylori* strains isolated from Western countries generally contains tandem EPIYA-A, EPIYA-B and EPIYA-C motifs, with a difference in the copies of the EPIYA-C motif. On the other hand, CagA in *H. pylori* strains isolated from East Asian countries consists of EPIYA-A, EPIYA-B and EPIYA-D motifs (Figure 1A) [30]. SHIP2-SH2 showed strong binding to phosphorylated EPIYA-C motif in a previous co-immunoprecipitation study, while it also bound to the phosphorylated EPIYA-D motif, although less strongly than to the EPIYA-C motif [22]. Once CagA binds to and tethers SHIP2 to the plasma membrane, SHIP2 triggers an increase in the PI(3,4)P2 level, which strengthens the attachment of *H. pylori* to gastric epithelial cells; this, then, increases the delivery of CagA into the host cells to further induce the epithelial–mesenchymal transition-like phenotype that usually reflects enhanced cell motility and cell invasion potential. So far, the interaction mechanism between SHIP2-SH2 and the EPIYA motifs of CagA from the aspects of structure, affinity and key residues remains unclear.

In recent years, and using NMR methods, we investigated the interaction of SHIP2-SH2 with Tyr-phosphorylated c-MET and FcgR2B, which mediate hepatocyte growth factor (HGF) and immunity signaling, respectively [13,21]. In this study, we further studied the interaction of SHIP2-SH2 with four types of Tyr-phosphorylated EPIYA motifs. NMR titrations confirmed that SHIP2-SH2 interacted with the four EPIYA motifs in vitro, but the affinity and interface of SHIP2-SH2 for the four motifs were quite different. The binding of EPIYA-A and EPIYA-B to SHIP2-SH2 was weak, with a dissociation constant (*K*_D_) of around 20 μM, and the binding interface of SHIP2-SH2 was limited to the pY-pocket and its surrounding area. Unexpectedly, the binding affinities of SHIP2-SH2 to EPIYA-C and EPIYA-D were similar, and the *K*_D_ values were around 3 μM, indicating an approximate 7-fold higher affinity than those to EPIYA-A/B. SHIP2-SH2 engaged a larger binding interface for EPIYA-C/D than for EPIYA-A/B, which included the pY-pocket and the area consisting of βE, βF and the EF-loop. These results were consistent with the previous study, which suggested that EPIYA-C/D are the dominant motifs preferentially binding to SHIP2 in vivo. By comparing our data for the binding of SHIP2-SH2 with Tyr-phosphorylated c-MET, FcgR2B and CagA, we found that SHIP2-SH2 displays selectivity to the aliphatic residues at the pY+3 positions. The preference order is Leu > Ile > Val, indicating that the longer the aliphatic side chain at this site, the higher the affinity for SHIP2-SH2. Our study improves the phosphorylated ligand-recognition mechanism of SHIP2-SH2 and provides important information for designing selective inhibitors for SHIP2.

## 2. Results

### 2.1. SHIP2-SH2 Binds with CagA EPIYA-C and EPIYA-D in a Similar Mode

Since CagA binds to SHIP2-SH2 mainly through the phosphorylated EPIYA-C and EPIYA-D motifs in vivo, and the EPIYA-C and EPIYA-D motifs share the same sequence (E-P-I-pY-A-T-I-D) in their 8-mer form (Figure 1B, −3 to +4), the interaction of SHIP2-SH2 with 8-mer EPIYA-CD phosphopeptide, which can represent both EPIYA-C and EPIYA-D in their 8-mer forms, was first investigated using NMR titration. When the phosphopeptide was titrated into ^15^N-labeled SHIP2-SH2, it can be clearly seen that numerous resonances shifted greatly in the ^1^H-^15^N HSQC spectra (Figure 1C). These results confirmed the binding between SHIP2-SH2 and phosphorylated EPIYA-C/D motifs. During the titration, most peaks from SHIP2-SH2 residues with significant chemical shift perturbations (CSPs) shifted successively from the free to completely-bound positions, which is characteristic of a fast chemical exchange on the NMR timescale. There were also a few peaks showing line broadening and decreased intensities during titration, representing intermediate chemical exchange behavior.

Our previous study indicated that 13-mer Tyr-phosphorylated FcgR2B-ITIM peptide binds to a larger SHIP2-SH2 interface than its 8-mer form. Thus, we performed NMR titration of ^15^N-labeled SHIP2-SH2 with 13-mer Tyr-phosphorylated EPIYA-C and EPIYA-D peptides to see whether this was also the case for CagA-EPYIA peptides. The results were largely similar to that of the titration with 8-mer EPIYA-CD phosphopeptide (Figure 1D,E). Meanwhile, when the ^1^H-^15^N HSQC spectra of SHIP2-SH2 bound by 13-mer EPIYA-C and EPIYA-D phosphopeptides were superimposed, most cross peaks overlapped very well (Figure 1F), and only four residues showed a chemical shift difference over 0.15 ppm, which indicated that EPIYA-C and EPIYA-D have a similar binding propensity to SHIP2-SH2.

**Figure 2:**
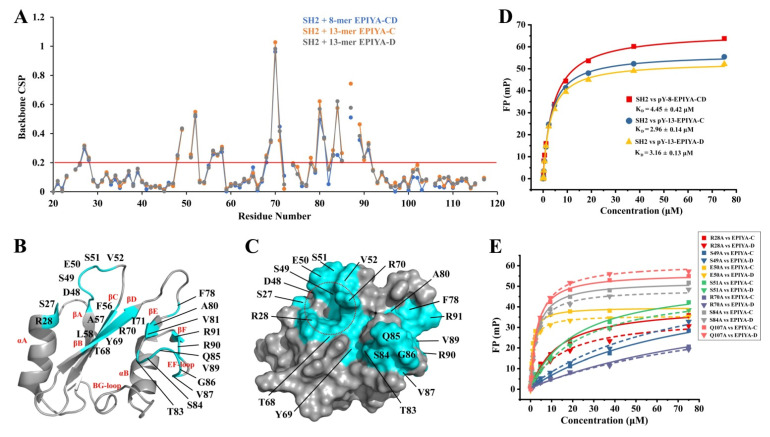
The binding interface and affinity of SHIP2-SH2 for CagA phosphopeptides from EPIYA-C and EPIYA-D motifs. (**A**) The chemical shift perturbation (CSP) of each residue during NMR titration with 8-mer and 13-mer CagA EPIYA-C and EPIYA-D phosphopeptides is shown with the threshold of 0.2 ppm for significant CSP, marked by the red line. (**B**,**C**) The residues with CSPs larger than 0.2 ppm or unassignable during the titration of 13-mer CagA EPIYA-C phosphopeptide are mapped onto ribbon representation (**B**) and molecular surface representation (**C**) of SHIP2-SH2 structure (PDB ID: 2MK2). Secondary elements are labeled in red font (**B**), and the pY-pocket is indicated by a red dashed circle (**C**). (**D**) Binding affinities of 8-mer EPIYA-CD, 13-mer EPIYA-C and 13-mer EPIYA-D phosphopeptides for SHIP2-SH2 revealed by fluorescence polarization (FP) assay. The fitted dissociation constants (*K*_D_, μM) are indicated as inset, respectively. (**E**) Key residues of SHIP2-SH2 for binding to 13-mer EPIYA-C and 13-mer EPIYA-D phosphopeptides revealed by mutagenesis and FP assay. The determined *K*_D_ values are shown in Table 1.

The binding interface of SHIP2-SH2 for EPIYA-C and EPIYA-D phosphopeptides was analyzed by calculating the CSP values of each SHIP2-SH2 residue. The results showed that the residues with a significant CSP (over 0.2 ppm, referring to the study of the interaction between SHIP2-SH2 and FcgR2B ITIM) were almost the same for the three phosphopeptides (Figure 2A), except F78 that only showed a CSP over 0.2 ppm in the titration of the 13-mer EPIYA-C phosphopeptide. When the residues with significant CSP values were mapped onto the structure of SHIP2-SH2, it was found that the binding interface mainly consisted of αA (S27 and R28), βB (D48), the BC-Loop (S49, E50, S51 and V52), βC (F56, A57 and L58), βD (T68, Y69, R70 and I71), the DE-loop (F78), βE (A80 and V81), the EF-Loop (T83, S84, Q85, G86, V87, V89, R90) and βF (R91) (Figure 2B). These residues were located in two regions on the SHIP2-SH2 surface, the pY-pocket and the hydrophobic pocket (Figure 2C), consistent with the basic mode for the binding of SHIP2-SH2 with its ligand, revealed by our previous studies. These data suggested that SHIP2-SH2 binds to EPIYA-C and EPIYA-D peptides with an identical interface at the significant CSP level of 0.2 ppm.

However, when comparing the CSP values in detail, it was found that there were still small differences between the CSPs for 8-mer and 13-mer peptides. A few residues showed greater CSPs in the binding of 13-mer EPIYA-C and EPIYA-D phosphopeptides than in the binding of 8-mer EPIYA-CD; these residues were mainly located in the βE (A80), EF-loop (Q82, S84 and V87), αB (L101, Y102 and A103) and BG-loop (Q107), although the CSPs for some of the residues were under 0.2 ppm. These suggest a mildly enhanced binding in this region after the phosphopeptide lengthened, which meant that the residues from pY+5 to pY+7 positions of EPIYA-C and EPIYA-D interact weakly with SHIP2-SH2. This was different from the case of FcgR2B-ITIM, where the residues from the SHIP2-SH2 BG-loop showed CSPs over 0.2 ppm after 13-mer FcgR2B-ITIM titration. The binding affinity of SHIP2-SH2 to different EPIYA-C and EPIYA-D phosphopeptides was subsequently characterized by fluorescence polarization (FP) experiment. The results showed that the *K*_D_ values of SHIP2-SH2 to 13-mer EPIYA-C and EPIYA-D were 2.96 μM and 3.16 μM, respectively, which are slightly lower than that to 8-mer EPIYA-CD (*K*_D_ = 4.45 μM) (Figure 2D). This indicates that the binding affinities of SHIP2-SH2 to 13-mer EPIYA-C and EPIYA-D are very close, but higher than to the 8-mer EPIYA-CD, which was consistent with the NMR titration data.

Further, alanine mutation-based analysis was performed to identify key SHIP2-SH2 residues for binding to EPIYA-C and EPIYA-D. Five residues in the pY-pocket, including R28, S49, E50, S51 and R70, and two residues in the electroneutral pocket, including S84 and Q107, were selected for mutagenesis, as referred to in the NMR titration data and the residues selected in our previous studies [13,21]. The binding affinities of these mutants for 13-mer CagA EPIYA-C and EPIYA-D phosphopeptides were determined by FP assay (Figure 2E). The *K_D_* values are summarized in Table 1. Among the mutants for the pY-pocket residues, R28A, S49A, S51A and R70A showed obvious reduced affinities for both 13-mer CagA EPIYA-C and EPIYA-D, suggesting that these residues are crucial for binding to the pY residue of EPIYA-C and EPIYA-D. E50A displayed decreased *K_D_* values for both EPIYA-C and EPIYA-D phosphopeptides, indicating increased affinities probably caused by reduced electrostatic repulsion, which was also found for Y1356-phosphorylated c-MET peptide and FcgR2B-ITIM phosphopeptide. For the two selected residues in the electroneutral pocket, the *K_D_* values for S84A and Q107A did not show a significant difference to WT for either EPIYA-C or EPIYA-D, which was similar to the case of c-MET but different to that of FcgR2B-ITIM phosphopeptide.

### 2.2. The SHIP2-SH2 EF- and BG-Loops Are Different from Those of SHP2 N-SH2

**Figure 3:**
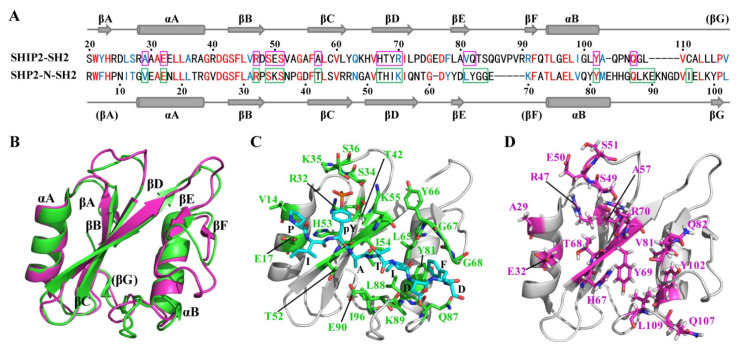
Comparison of the sequence and structure of SHIP2-SH2 and human SHP2 N-SH2. (**A**) Sequence alignment of SHIP2-SH2 and SHP2 N-SH2. The identical residues are colored in red, while similar residues are in blue. Secondary structural elements are marked on top and bottom, respectively, for SHIP2-SH2 and SHP2 N-SH2, and those in brackets show loop structure in corresponding structures. The SHP2 N-SH2 residues labeled by green boxes show contact with the CagA EPIYA-D phosphopeptide in the complex structure (PDB ID: 5X94), while the corresponding residues in SHIP2-SH2 are labeled by magenta boxes. (**B**) Superimposition of the structures of SHIP2-SH2 (magenta, PDB ID: 2MK2) and SHP2 N-SH2 (green, PDB ID: 5X94, chain A). (**C**) The complex structure of SHP2 N-SH2 binding with the CagA EPIYA-D phosphopeptide (PDB ID: 5X94). The SHP2 N-SH2 residues in contact with CagA EPIYA-D phosphopeptide are shown in sticks and colored in green, while the residues belonging to EPIYA-D are shown by cyan sticks. (**D**) The structure of apo SHIP2-SH2 with the residues corresponding to the EPIYA-D-interacting SHP2 N-SH2 residues shown by magenta sticks.

CagA EPIYA-C and EPIYA-D motifs can not only interact with SHIP2, but also with SHP2 (SH2 domain-containing phosphotyrosine phosphatase 2). SHP2 has two tandem SH2 domains, N-SH2 and C-SH2. Both N-SH2 and C-SH2 displayed a higher affinity to EPIYA-D than to EPIYA-C phosphopeptide in vitro, and N-SH2 exhibited a higher affinity to the two peptides than did C-SH2. A structural study revealed that a groove formed between the EF- and BG-loops can accommodate the hydrophobic Phe residue at the pY+5 position of EPIYA-D to achieve a higher affinity than that to EPIYA-C, which has an Asp residue at the pY+5 position [26]. However, SHIP2-SH2 exhibited a similar affinity to EPIYA-D and EPIYA-C phosphopeptides in our study. In order to explain the different bindings of SHIP2-SH2 and SHP2–SH2 to the EPIYA-C and EPIYA-D, we carried out sequence and structure alignment between SHIP2-SH2 and SHP2 N-SH2. The sequence similarity between SHIP2-SH2 and SHP2 N-SH2 was 43.9% (Figure 3A), and their overall structures were highly similar (Figure 3B). The residues consisting of the hydrophobic pocket for binding to pY+1 (Ala) and pY+3 (Ile) residues of EPIYA-D are similar. T52, I54, L65, Y81 and L88 of SHP2 N-SH2 correspond to H67, Y69, V81, Y102 and L109 of SHIP2-SH2. A major difference between the two was located in the EF- and BG-loops. The flexible EF-loop of SHP2 N-SH2 only has four residues (YGGE), while SHIP2-SH2 has nine residues (QTSQGVPVR). The flexible region of the BG-loop of SHP2 N-SH2 has eleven residues (HHGQLKEKNGD), while that of SHIP2-SH2 only has five residues (QPNQG) (Figure 3A). These should contribute to the different properties of SHIP2-SH2 and SHP2 N-SH2 binding to the EPIYA-C and EPIYA-D. Detailed comparison of the residues for binding to EPIYA-D suggest that the T42 to A57, T52 to H67, and H53 to T68 substitutions may also contribute to the different binding properties of the two SH2 domains (Figure 3C,D). Briefly, the different sequences and structures of the EF- and BG-loops should make SHIP2-SH2 unable to selectively bind with the EPIYA-C or EPIYA-D, as is the case in SHP2 N-SH2. Accordingly, the NMR titration data revealed no significant CSP in the BG-loop for either 13-mer EPIYA-C or EPIYA-D phosphopeptides.

Previous study evidenced the ability of SHIP2-SH2 to directly interact with CagA EPIYA motifs using in-cell methods such as Co-IP. By using different combinations of EPIYA motifs, including A+B, A+B+C and A+B+D in the Co-IP assay, it was found that SHIP2-SH2 binds the strongest to EPIYA-(A+B+C), less strong to EPIYA-(A+B+D) and the weakest to EPIYA-(A+B), suggesting that SHIP2-SH2 binds to these motifs with a preference of C > D > A/B [22]. In our study, although EPIYA-C caused more remarkable CSPs in a small number of residues in NMR titration, the *K*_D_ values determined by FP for 13-mer EPIYA-C and EPIYA-D phosphopeptides were not significantly different. This divergence of binding affinity determined by Co-IP and FP may be due to the CagA used in Co-IP being full length, while in FP it was a short peptide. As the sequence difference was not limited to the EPIYA motifs, the sequence adjacent to the EPIYA-C motif may provide additional binding of SHIP2. Moreover, the phosphorylation level of individual EPIYA motifs of CagA may not be identical in cell, which would affect the binding of SHIP2. Whatever the reasons for the difference between in vitro and in vivo results in terms of 13-mer EPIYA-C and EPIYA-D phosphopeptides, SHIP2 showed no obvious difference in binding affinity, which was different to the case of SHP2–SH2s.

### 2.3. SHIP2-SH2 Binds to EPIYA-A and EPIYA-B with Weaker Affinity Than to EPIYA-C and EPIYA-D

**Figure 4:**
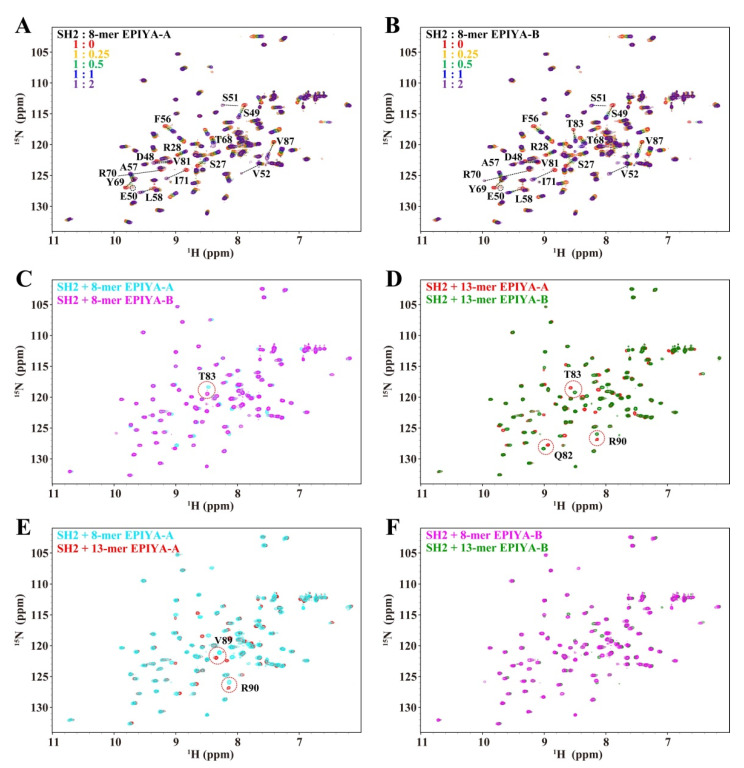
SHIP2-SH2 interacts with Tyr-phosphorylated CagA EPIYA-A and EPIYA-B motifs. NMR titrations of SHIP2-SH2 by 8-mer Tyr-phosphorylated CagA EPIYA-A (**A**) and EPIYA-B (**B**) peptide. The residues with remarkable chemical shift perturbations are labeled. (**C**) Superposed spectra of SHIP2-SH2 bound by Tyr-phosphorylated 8-mer EPIYA-A and EPIYA-B peptides. (**D**) Superposed spectra of SHIP2-SH2 bound by Tyr-phosphorylated 13-mer EPIYA-A and EPIYA-B peptides. (**E**) Superposed spectra of SHIP2-SH2 bound by Tyr-phosphorylated 8-mer and 13-mer EPIYA-A peptides. (**F**) Superposed spectra of SHIP2-SH2 bound by Tyr-phosphorylated 8-mer and 13-mer EPIYA-B peptides. The residues with chemical shift differences larger than 0.15 ppm are highlighted by red circles.

Although SHIP2 showed more preference to bind with EPIYA-C and EPIYA-D in cell, a weak binding to EPIYA-A and EPIYA-B can also be found. Thus, we further investigated the binding mechanism of SHIP2-SH2 to EPIYA-A and EPIYA-B, using similar methods for studying EPIYA-C and EPIYA-D. NMR titrations of SHIP2-SH2 with 8-mer EPIYA-A and EPIYA-B phosphopeptides were first performed (Figure 4A,B). It could be clearly seen that, along with the addition of phosphopeptide, many SHIP2-SH2 peaks shifted progressively in the ^1^H-^15^N HSQC spectra of the two titrations, evidencing the ability of Tyr-phosphorylated EPIYA-A and EPIYA-B to bind to SHIP2-SH2. The continuous shift of the peaks in both titration experiments showed the binding to be in the fast exchange regime on the NMR timescale. When the two titrated HSQC spectra were superimposed, most peaks were well overlapped, indicating that the binding mode of SHIP2-SH2 for the two phosphopeptides is similar (Figure 4C). The interaction of 13-mer EPIYA-A and EPIYA-B phosphopeptides with SHIP2-SH2 was further tested using NMR titration. According to the NMR spectra, the 13-mer phosphopeptides of EPIYA-A and EPIYA-B also bind to SHIP2-SH2 and differ little from each other (Figure 4D) or from their respective 8-mer forms (Figure 4E,F). The spectra were well overlapped and there was almost no residue with a chemical shift difference greater than 0.15 ppm. These results indicate that the additional residues of 13-mer EPIYA-A and EPIYA-B do not have, or have pretty weak, interactions with SHIP2-SH2 compared with 8-mer forms.

**Figure 5:**
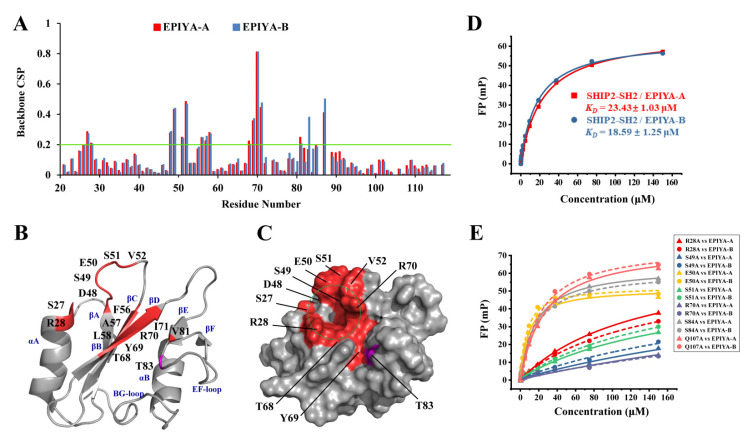
The binding interface and affinity of SHIP2-SH2 for CagA phosphopeptides from EPIYA-A and EPIYA-B motifs. (**A**) The chemical shift perturbation (CSP) of each residue during NMR titration with 8-mer CagA EPIYA-A and EPIYA-B phosphopeptides is shown with the threshold of 0.2 ppm for significant CSP marked by a green line. (**B**,**C**) The residues with CSPs larger than 0.2 ppm during the titration of 8-mer CagA EPIYA-A and EPIYA-B phosphopeptide are mapped onto ribbon representation (**B**) and molecular surface representation (**C**) of SHIP2-SH2 structure (PDB ID: 2MK2). Secondary elements are labeled in blue font (**B**), and the pY-pocket is indicated by a green dashed circle (**C**). (**D**) Binding affinities of 13-mer EPIYA-A and 13-mer EPIYA-B phosphopeptides for SHIP2-SH2 revealed by FP assay. The fitted dissociation constants (*K*_D_, μM) are indicated as inset, respectively. (**E**) Key residues of SHIP2-SH2 for binding to 13-mer EPIYA-A and 13-mer EPIYA-B phosphopeptides revealed by mutagenesis and FP assay. The determined *K*_D_ values are shown in Table 1.

The chemical shift perturbation data of the NMR titrations were quantified and mapped onto the SHIP2-SH2 structure to obtain the detailed binding information (Figure 5A–C). The significantly disturbed amino acid residues with a CSP greater than 0.2 ppm during titrations of EPIYA-A and EPIYA-B phosphopeptides were mainly located in the pY-pocket region on the surface of SHIP2-SH2. The binding interface for EPIYA-A was formed by αA (S27 and R28), βB (D48), the BC-Loop (S49, E50, S51 and V52), βC (F56, A57 and L58), βD (T68, Y69, R70 and I71) and βE (V81). Additionally, the binding interface for EPIYA-B included T83 in the EF-loop. These results indicate that the binding interface of SHIP2-SH2 for the two phosphopeptides was identical. The affinities for the interaction of SHIP2-SH2 with 13-mer EPIYA-A and EPIYA-B phosphopeptides were subsequently determined by FP. The *K*_D_ value of SHIP2-SH2 binding to EPIYA-A was 23.43 μM, and that to EPIYA-B was 18.59 μM, indicating that the two EPIYA motifs bind to SHIP2-SH2 with basically the same affinity (Figure 5D). The similar affinity and binding interface strongly suggest that SHIP2-SH2 binds with EPIYA-A and EPIYA-B in the same binding mode. EPIYA-A and EPIYA-B share the same residues at their pY+1 (Ala) and pY+3 (Val) sites (Figure 1B), which are thought to be critical for SHIP2-SH2 to recognize pY-peptide. Therefore, this may also be responsible for SHIP2-SH2 sharing the similar binding mode of the two motifs.

Subsequently, the seven mutants of SHIP2-SH2, i.e., R28A, S49A, E50A, S51A, R70A, S84A and Q107A, were tested for the binding with 13-mer CagA EPIYA-A and EPIYA-B phosphopeptides using FP assay, to see whether these residues are important for the binding of EPIYA-A and EPIYA-B (Figure 5E, Table 1). Similar to the situation of EPIYA-C and EPIYA-D, R28A, S49A, S51A and R70A showed obvious reduced affinities for both EPIYA-A and EPIYA-B, suggesting that these residues are crucial for binding to the pY residue of EPIYA-A and EPIYA-B. E50A also displayed decreased *K_D_* values for both EPIYA-A and EPIYA-B. The *K_D_* values of S84A and Q107A did not show a significant difference to the WT for EPIYA-B. The *K_D_* value of Q107A did not show a significant difference to the WT for EPIYA-A, but S84A showed slightly increased affinity to EPIYA-A, which was similar to the case of FcgR2B-ITIM phosphopeptide.

EPIYA-A and EPIYA-B showed a weak SHIP2-SH2 binding ability, which was often overlooked in previous in-cell experiments. Since their affinities are obviously weaker than those of EPIYA-C and EPIYA-D, a preference of SHIP2 for EPIYA-C or EPIYA-D is a matter of course. However, when some mutations occur in EPIYA-C or EPIYA-D, CagA may retain part of its ability to bind to SHIP2 through the binding mediated by EPIYA-A or EPIYA-B, and thus continue to recruit SHIP2 to form complexes and function in an unconventional weak interaction mode. On the other hand, in our previous study, SHIP2 was suggested to have the potential to form dimers through a coiled-coil domain adjacent to the SH2 domain [14]. In this case, the interaction between CagA and SHIP2 in cell may include a simultaneous binding of two Tyr-phosphorylated EPIYA motifs by a SHIP2 dimer, which may potentially enhance the binding of EPIYA motifs, even EPIYA-A and EPIYA-B.

### 2.4. The Rule in the Binding Affinity and Interface of SHIP2-SH2 for Different Natural Ligands

**Figure 6:**
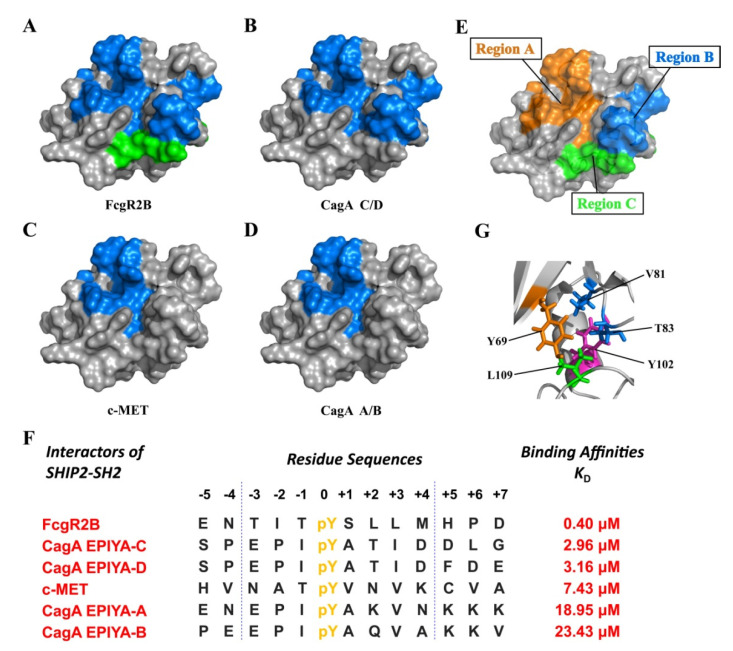
The binding interface and affinity of SHIP2-SH2 for different native ligands. The binding interface of SHIP2-SH2 for FcgR2B ITIM (**A**), CagA EPIYA-C/D (**B**), c-MET C-terminus (**C**), CagA EPIYA-A/B (**D**) is colored in blue. The additional interface of SHIP2-SH2 for 13-mer FcgR2B ITIM compared to 8-mer FcgR2B ITIM is colored in green. The interface was drawn according to the residues of SHIP2-SH2 showing a CSP greater than 0.2 ppm in NMR titration experiments with respective ligand. (**E**) The summarized three regions of SHIP2-SH2 which are differently engaged for binding different ligands are highlighted. (**F**) The summarized sequences and affinities of different SHIP2-SH2 ligands. (**G**) The potential pocket of SHIP2-SH2 for the pY+3 residue in ligand. Potential residues for interacting with the pY+3 residue are shown with sticks and labeled in color, similar to (**E**). Y102 at bottom of the pocket is colored in magenta.

Although the pY-motifs in the native interaction partners of SHIP2-SH2 identified to date show low sequence similarity, a consensus sequence of pY[S/Y][L/Y/M][L/M/I/V] for the high-affinity ligand peptides of SHIP2-SH2 was defined through the screening of a synthesized pY peptide library [20]. SHIP2-SH2 plays an important role in the recognition of pY-related signaling pathways. To date, SHIP2 was reported to be involved in many cellular signaling pathways and to recognize a variety of natural proteins containing pY through SH2, which include FcRL6, FcgR2B, FcgR2A, c-MET/HGFR, CagA and N-WASP, etc. We studied the binding of SHIP2-SH2 with c-MET, FcgR2B and CagA using NMR and FP. By combining and analyzing our data, we found that the binding interface and affinity of SHIP2-SH2 for these ligands vary significantly (Figure 6A–E). CagA EPIYA-A/B and c-MET displayed low affinity and their interfaces were mainly located in the pY-pocket and a small part of the hydrophobic pocket (region A). CagA EPIYA-C/D exhibited a moderate affinity and caused more CSP in the EF-loop (region B), suggesting that they may bind more strongly at the hydrophobic pocket. FcgR2B binds with high affinity and causes additional CSP in the BG-loop (region C), implying that the residues following pY+3 create additional binding to SHIP2-SH2.

When the natural phosphopeptides interacting with SHIP2-SH2 were ranked according to their affinities to SHIP2-SH2, it was seen that the affinity was related to the residue type at the pY+3 position (Figure 6F). The ligands showing high and moderate affinities have Leu (L) or Ile (I) residues at this site, while ligands with a low affinity have Val (V) at this site. These indicate that SHIP2-SH2 may have a preference for the hydrophobic side chain of the residue at the pY+3 site of its Tyr-phosphorylated ligand. The preference order is L > I > V, suggesting that the longer the aliphatic side chain of the residues, the higher the affinity for SHIP2-SH2. Through the structure alignment with SHP2 N-SH2 (Figure 3C,D), we deduced that the electroneutral region of SHIP2-SH2 formed by βD, βE, αB, and the EF-loop and BG-loop can bind to the pY+1 and pY+3 residues of ligands, and the sites for binding with the pY+3 residue potentially include Y69, V81, T83, Y102 and L109, most of which are hydrophobic residues (Figure 6G). Because the longer aliphatic side chain can stretch deeper into the pocket and make more hydrophobic contacts, the high-affinity ligand of SHIP2-SH2 should have Leu at the pY+3 site. A previous study using surface plasmon resonance (SPR) determined the affinities of 6-mer FcgR2B ITIM (-2 to +3, ITpYSLL) and FcgR2A ITAM (−2 to +3, GGpYMTL) phosphopeptides for SHIP2-SH2, which both have Leu at the pY+3 position, and found that they have comparable *K*_D_ values [20], supporting the rule we propose here. Meanwhile, the type of residues following pY+3, especial the pY+5 residue, should also be optimized to achieve higher affinity (perhaps referring to the sequence of FcgR2B). Further determination of the structure of SHIP2-SH2 in complex with its high-affinity ligand will provide more detailed information to design a competitive inhibitor for SHIP2-SH2.

## 3. Materials and Methods

### 3.1. Sample Preparation

Expression and purification of wild-type SHIP2-SH2 and its mutants including R28A, S49A, E50A, S51A, R70A, S84A and Q107A were carried out using the previously described method [13], and circular dichroism (CD) spectra were collected to confirm no obvious secondary structure change of the mutants. The purified proteins were concentrated to a final concentration of 0.2 mM for NMR experiments in the NMR buffer containing 90% H_2_O/10% D_2_O (*v*/*v*), 20 mM Tris, 100 mM NaCl, 10 mM DTT and 0.02% NaN_3_ at pH 7.5. Peptides derived from the four types of EPIYA motifs were synthesized by GL Biochem Ltd. (Shanghai, China). Tyr-phosphorylated peptides included: 8-mer (E-P-I-pY-A-K-V-N) and 13-mer (E-N-E-P-I-pY-A-K-V-N-K-K-K) EPIYA-A; 8-mer (E-P-I-pY-A-Q-V-A) and 13-mer (P-E-E-P-I-pY-A-Q-V-A-K-K-V) EPIYA-B; and 8-mer EPIYA-CD (E-P-I-pY-A-T-I-D), 13-mer EPIYA-C (S-P-E-P-I-pY-A-T-I-D-D-L-G) and 13-mer EPIYA-D (S-P-E-P-I-pY-A-T-I-D-F-D-E). The purities of the peptides were determined to be above 98% by high performance liquid chromatography (HPLC) and the molecular weights were confirmed by electrospray ionization mass spectrometry (ESI-MS).

### 3.2. NMR Titration

NMR titration experiments were performed at 298 K using a Bruker Avance III 600 MHz instrument. Different CagA EPIYA peptides were added to the sample solution containing 0.2 mM ^15^N-labeled SHIP2-SH2, respectively. The concentration of stock solutions for all peptides dissolved in the NMR buffer was 4 mM. Before NMR data collection, the samples containing the corresponding peptide and SHIP2-SH2 were mixed and allowed to equilibrate for over 1 h. In the titration of SHIP2-SH2 with EPIYA-C/D, ^1^H-^15^N HSQC spectra of SHIP2-SH2 mixed with peptide at molar ratios of 1:0, 1:0.25, 1:0.5, 1:0.75, 1:1, 1:1.25, 1:5 and 1:2 were collected, respectively. In the titration of SHIP2-SH2 with EPIYA-A/B, ^1^H-^15^N HSQC spectra of SHIP2-SH2 mixed with peptide at molar ratios of 1:0, 1:0.25, 1:0.5, 1:0.75, 1:1, 1:2 and 1:4 were collected, respectively. For clarity, the spectra are not all shown in Figure 1 and Figure 4. The chemical shift assignments for free-state SHIP2-SH2 were based on our previous study, and the chemical shifts for SHIP2-SH2 in the peptide-bound state were assigned in this study. The equation used for calculating chemical shift perturbations (CSPs) was the same as described in the previous study [13], while CSP values greater than 0.2 ppm were considered to be significant.

### 3.3. Chemical Shift Assignments

The NMR data for backbone chemical shift assignments for SHIP2-SH2 in complex with 8-mer Tyr-phosphorylated CagA EPIYA-CD peptides were collected on a Bruker Avance III 600 MHz spectrometer at 298 K, and included 2D ^1^H-^15^N HSQC, and 3D HNCA, HNCO, HN(CO)CA, HNCACB and CBCA(CO)NH. The backbone chemical shifts for SHIP2-SH2 bound by 8-mer EPIYA-CD peptides were firstly assigned by tracing the peak change during titration, and then manually validating and correcting them according to the collected 3D-NMR data. The chemical shift assignments were 97% complete for backbone HN cross peaks of SHIP2-SH2 bound by the 8-mer EPIYA-CD peptide, based on which the backbone chemical shifts of SHIP2-SH2 bound by the 13-mer EPIYA-C and EPIYA-D peptides were subsequently assigned. As the backbone chemical shifts of SHIP2-SH2 mixed with the EPIYA-A and EPIYA-B peptides at different molar ratios showed successive migration of the HN cross peaks, the backbone chemical shifts in the peptide-bound state were quickly assigned based on the assignments of free-state SHIP2-SH2. In the titration of SHIP2-SH2 with each phosphopeptide, the bound-state resonances for residues E50 and G86 were untraceable and not well determined in each ^1^H-^15^N HSQC spectrum, so the two residues were not assigned.

### 3.4. Fluorescence Polarization

The used phosphopeptides for different versions of CagA EPIYA A/B/C/D motifs were labeled with FAM at the N-terminal end and dissolved in a buffer containing 20 mM Tris, 100 mM NaCl, 10 mM DTT and 0.02% NaN_3_, at pH 7.5. The peptide concentration was kept at 100 nM, while wild-type SHIP2-SH2 and its mutants were serially diluted with the concentrations ranging from micromolar to nanomolar, and then mixed with the peptide, respectively. FP values of FAM-labeled peptides bound by SHIP2-SH2 were measured using a SpectraMax i3x multi-mode plate reader (Molecular Devices) by recording the excitation at 485 nm and emission at 528 nm. Each binding reaction was repeated three times, and the polarization values were averaged (given in units of mP). The averaged polarization value of each titration point was subtracted from that of the free peptide to obtain the final value. The dissociation constants (*K*_D_) were calculated using the final polarization value of each titration point as previously described [13].

## 4. Conclusions

Stomach cancer is the third most common cancer in the world, and is most often caused by the CagA-positive *H. pylori*. After CagA enters host cells, several EPIYA motifs at the CagA C-terminal undergo tyrosine phosphorylation and interact with a series of proteins containing the SH2 domain to induce cell carcinogenesis and resist immune surveillance. In this process, the interaction between SHIP2-SH2 and CagA plays a crucial role in inducing cell tumorigenesis, but there was a lack of studies on the interaction mechanism thus far. In this paper, the interaction mechanism between the SHIP2-SH2 domain and four different tyrosine-phosphorylated EPIYA motifs (EPIYA-A, EPIYA-B, EPIYA-C and EPIYA-D) in CagA was studied by NMR titration and fluorescence polarization. The results show that EPIYA-C and -D bind to a larger SHIP2-SH2 interface with similar and strong affinity, while EPIYA-A and -B bind to a smaller interface with weak affinity. Considering that SHIP2 may dimerize through its coiled-coil domain in vivo, we speculate that *H. pylori* can engage varied combinations of EPIYA motifs for hijacking SHIP2, such as binding to SHIP2 monomers through the high-affinity EPIYA-C or -D, or binding to SHIP2 dimers through the low-affinity EPIYA-A and -B, simultaneously. Thus, the *H. pylori* strains from Western countries and East Asian countries may both be able to hijack SHIP2 to induce disease. Meanwhile, by analyzing the binding patterns of SHIP2-SH2 to four phosphorylated EPIYA- polypeptides and comparing them with c-MET and FcgR2B, the amino acid sequences of these natural ligands can be summarized and classified. The results show that SHIP2-SH2 very likely has a selective preference for the aliphatic side chain of the residues at the pY+3 position in the ligand. Through the characterization of *K*_D_ values, it was found that the order of residue preference was L > I > V. This paper extended the understanding that SHIP2-SH2 uses different regions to selectively recognize pY-ligands from different signaling pathways; it also explored the rule of ligand sequence for SHIP2-SH2 binding, which lays a foundation for the in-depth understanding of the selectivity and specificity of the SHIP2 function. In addition, it provides help for the design of polypeptide inhibitor drugs against *H. pylori*.

## Figures and Tables

**Table 1:** The dissociation constants (*K*_D_, μM) of wild-type (WT) and seven mutants of SHIP2-SH2 for four types of 13-mer EPIYA peptides determined by fluorescence polarization assays.

SHIP2-SH2	EPIYA-A	EPIYA-B	EPIYA-C	EPIYA-D
Wild type	23.43 ± 1.03 ^a^	18.59 ± 1.25	2.96 ± 0.14	3.16 ± 0.13
R28A	132.69 ± 6.95	142.58 ± 17.43	12.87 ± 1.38	15.54 ± 2.77
S49A	418.55 ± 18.59	302.74 ± 16.24	77.54 ± 3.12	76.82 ± 5.16
E50A	8.28 ± 0.67	6.60 ± 0.60	0.96 ± 0.14	1.14 ± 0.16
S51A	180.00 ± 37.36	170.76 ± 35.96	22.38 ± 2.88	24.55 ± 2.23
R70A	539.26 ± 24.19	532.45 ± 47.48	119.27 ± 4.93	116.08 ± 6.61
S84A	16.44 ± 0.47	17.92 ± 0.95	2.79 ± 0.18	2.69 ± 0.19
Q107A	25.53 ± 1.50	21.45 ± 1.33	2.78 ± 0.14	3.46 ± 0.20

^a^ The standard errors were generated during the fitting of the binding curves.

## Data Availability

Data supporting the reported results will be available from the corresponding authors (Jiang Zhu and Yunhuang Yang).
